# Effects of noise intensity on concentration levels of chainsaw operators and harvesting workers in industrial forest plantation, North Sumatera, Indonesia

**DOI:** 10.12688/f1000research.114592.3

**Published:** 2024-01-04

**Authors:** Muhdi Muhdi, Diana Sofia Hanafiah, Asmarlaili Sahar, Alex Angenano Telaumbanua

**Affiliations:** 1 Faculty of Forestry, Universitas Sumatera Utara, Medan, 20155, Indonesia; 2 Faculty of Agriculture, Universitas Sumatera Utara, Medan, 20155, Indonesia

**Keywords:** Chainsaw, forest harvesting, industrial forest plantation, noise

## Abstract

**Background:**

Noise has the potential to affect the comfort and health of workers. The objective of this research was to determines the effects of noise on the concentration levels of both chainsaw operators and harvesting workers in industrial forest plantation, North Sumatra, Indonesia.

**Methods:**

This experimental study included 20 respondents which consisted of 10 chainsaw operators/helpers and 10 harvesting workers. All respondents were exposed to the sound of a chainsaw in three different conditions (idle, half gas and racing conditions) with and without personal protective equipment (PPEs: earmuffs, ear plugs and without PPE). The sound intensity produced by the chainsaw and the noise received by the respondents were measured using a sound level meter. Respondents’ perception toward noise was recorded using a Likert scale. Respondents’ concentration level was assessed by giving 25 math-questions to be answered within 10 minutes. Wilcoxon sign rank test was used to analysed paired data.

**Results:**

The average sound intensity received by respondents’ left and right ears were lower than the average sound intensity produced by the chainsaw. The use of earmuffs leads to better perception towards noise when compared with the use of earplugs and the absence of any PPE. Based on Wilcoxon test, the noise did not have significant impact on the concentration level of chainsaw operators, whilst the contrary result is true for harvesting workers.

**Conclusions:**

The research indicated that although the noise produced by the chainsaw was considered noisy for both chainsaw operators and harvesting workers, it did not have a significant effect on the concentration level of chainsaw operators and only affected the harvesting workers. Therefore, given that the harvesting workers were still affected by the noise, noise control measures are still needed to ensure occupational safety and health for the workers.

## Introduction

The timber harvesting process is considered a strenuous activity due to the overall forest harvesting processes, facilities, and infrastructure as well as natural factors such as topography and climate, which are complex entities that must be well-directed and well organized. ^1^^,^^2^ In addition, forestry workers are at high-risk for accident and health problems. Accidents may happen due to several factors, such as carelessness of workers, inadequate skill or lack of occupational experience to operate heavy equipment, and low awareness towards occupational health and safety aspect. ^3^^,^^4^

Despite technological advances that have resulted in a diverse range of timber harvesting machinery, ^5^ chainsaws are still widely used in forest operation due to their multifunctional use and low financial investment. ^6^ On the other hand, the use of chainsaws has been linked to the high accident rates in professional and non-professional work. ^7^^,^^8^

Chainsaw operators are undoubtly exposed to some threats such as noise, hand-arm vibrations (HAVs), exhaust gases, and timber dust. ^9^^,^^10^ According to Nugroho, ^11^ chainsaws are more dangerous than ordinary saws in many ways. A rotating chain can cause serious injury and noise produced by the machine could interfere with hearing in communication. In fact, continuous exposures to noise can cause health problems and discomfort at work, ranging from physiological and psychological disorders, balance disorders, communication difficulties, to hearing loss. ^12^ Physiological disorders that may occur in response to noise are raised blood pressure and heart rate, reduced hearing acuity, earaches, nausea, impaired muscle control, and others. ^13^ A study also found that noise (up to 85 dBA) could induce stress in some people. ^14^ It is also widely known that exposure to noise that exceed the allowed threshold could pose the operator to the risk of hearing loss. ^15^ Together, noise and its subsequent effects on health and safety aspect could lead to the decrease in employees’ work performance. ^12^ In addition, noise can also cause mental disturbances such as increased irritability, anxiety and impaired concentration which could lead to safety hazard. ^16^ Therefore, the noise that exceeds the allowed threshold and lasts for a long time must be controlled or prevented so as not to interfere with human life.

The purpose of noise control is to prevent workers from being exposed to these occupational hazards. This can be done by several methods, ranging from the use of personal protective equipment and implementation of rotational shiftwork, to the substitution or elimination of the noise source. ^17^ Protective strategies also include identification of noise problems in workplace and determination of noise levels received by employees. ^15^ According to Chandra, ^18^ the main tool for measuring noise levels is using a sound level meter. This tool works to measure noise in the range of 30 to 130 decibels (dB) with frequencies between 20 to 20,000 Hertz (Hz). The result of this measurements are then compared with the threshold value. In Indonesia, to protect the safety and health of workers, the government has also issued various policies related to the threshold value of noise standard (including Decree of the Minister of Manpower No. Kep-51/MEN/1999 on Physical Threshold Values at Work Sites). The government has also adopted the logging work standards formulated by the International Labour Organization. ^19^

Given that noise plays a significant role in determining occupational safety and health, research on the noise intensity and its control efforts, as well as its impact on the perception and concentration of workers in a workplace with significant noise become mandatory. This research was conducted to measure workers’ perception of noise generated by chainsaws in logging activities, and to determines the effects of noise on the concentration levels of both chainsaw operators and harvesting workers in industrial forest plantation, North Sumatra, Indonesia. The result of this study is important in order to reduce noise exposure by continuing to innovate, improve technology, remodify, and other aspects needed in an industrial forest plantation.

## Methods

### Ethical consideration

All procedures in this study were approved by the Health Research Ethics Committee at the Faculty of Medicine, Universitas Sumatera Utara, Medan, North Sumatra, Indonesia (14 ^th^ March 2021). Written informed consent was obtained from each respondent following the explanation about the nature of the study.

### Target groups

The target groups in this study was all workers at PT. Toba Pulp Lestari, North Sumatera, Indonesia. The sampling technique used in this study was non-probability sampling using a saturated sampling method. Inclusion criteria for this study were (1) chainsaw operator or harvesting workers who has worked for at least a year, (2) allowed by the supervisor to participate in the study, (3) right-handed. Left-handed workers were excluded from the study because the factor that affects the difference in sound intensity received by the left ear and the right ear is the anthropometry of the normal respondent's body (right-handed). In addition, the sound intensity received by the left ear is greater than that of the right ear due to the distance between the sound source (chainsaw) which is closer to the left ear than to the right ear. To maintain work productivity, respondents were chosen from different compartments/team (one respondent/team). A total of 20 workers were recruited as respondents, which comprised of 10 chainsaw operators and 10 harvesting workers.

### Study design and groups

The study conducted was an experimental study with pre- and post-test group design. Respondents were grouped into chainsaw operator group and harvesting workers group. Each group consisted of 10 workers. All respondents in each group were exposed to the sound of a real chainsaw in three different conditions, namely idle (gas trigger was not pulled), half gas (gas trigger was half pulled) and racing (gas trigger was fully pulled). During the exposure, sound intensity measurement was done in the chainsaw machine and workers’ left and right ear with three replication with a span of 30 seconds. The procedures took place in an outdoor setting at the timber harvesting site.

After that, respondents’ perception toward the noise produced by the chainsaw was measured three times in each condition (idle, half gas, racing) by using Likert scale. First measurement was done without the use of any personal protective equipment. In the second measurement, respondents were asked to use the earmuff. Lastly, in the third measurement, the respondents were asked to use the earplug. So that in total, for the measurement of respondents’ perception toward noise, each respondent was exposed to the noise of chainsaw nine times (3 times during idle conditions, 3 times during half gas conditions, and 3 times during racing conditions).

Respondents’ concentration level was later assessed using a designated questionnaire (detailed below) after the exposure of the sound of chainsaw in idle and racing conditions only. The measurements in each condition were done three times; without the use of any personal protective equipment, after using earmuff, and after using earplug. In this step, the respondent was exposed to the noise six times (3 times during idle conditions, and 3 times during racing conditions).

Since all measurements were done in outdoor setting, there were potential source of bias, such as the present of noise coming from other chainsaws or machinery used in the work setting. In order to minimize this bias, we decided to do the research during resting time (12.00-13.00 Western Indonesian Time), so that there were no other concurrent activities that may distract or produced extra noise that may affect the measurements. According to previous study, during this range of time, healthy individual also showed acceptable levels of cognitive performance. This may reduce the effect of circadian rhythm variations in each respondents while performing the test, especially while measuring the concentration level.

### Respondent interview

Interviews were conducted to gather information about respondents’ age, working experience expressed in years, and whether they were frequently exposed to noise outside working environment. The structured-interview was done by Associate of Accounting Technicians (AAT) at the logging site in PT. Toba Pulp Lestari for approximately 5 minutes. The interview guide was developed by the authors to include all necessary data and no prior testing was done. ^20^ The data were recorded in a dummy table that has been prepared prior to the interview. No audio/video were recorded.

### Measurement of sound intensity

Sound intensity was measured in decibels (dB) using a sound level meter (Danoplus SLM-25, Danoplus, China) for ten minutes. Measurements were made on the chainsaw machine (Husqvarna 365, Husqvarna AB, Stockholm) ( Table 1) and respondents’ right and left ear. The respondents were asked to stand up and place the chainsaw according to their usual working state. Sound intensity measurement on the chainsaw machine was done by placing the sound level meter approximately 5-10 cm from the chainsaw. Meanwhile, measurements in the right and left ear were done by placing sound level meters (one on the right ear, and one in left ear) in direct contact with the ear. All three measurements were done at the same time using different sound level meter.

**Table 1.** Chainsaw specifications.

**Engine**	Output power	**4.8 kW**
	Cylinder displacement	**93.6 cm** ^**3**^
	Electrode gap	**0.5 mm**
	Cylinders	**1**
	Number of strokes	**2-stroke engine**
**Cutting equipment**	Cutting depth, max	**400 mm**
	Blade thickness, max	**5.7 mm**
	Peripheral speed, max	**26 m/s**
**Dimensions**	Product size length	**846 mm**
	Product size width	**272 mm**
	Product size height	**370 mm**
	Weight	**9.7 kg**
**Sound and noise**	Sound power level, guaranteed (LWA)	**125 dB(A)**
	Sound pressure level at operators ear	**108 dB(A)**
**Vibrations**	Vibrations left/front handle	**3.6 m/s** ^**2**^
	Vibrations right/rear handle	**4.7 m/s** ^**2**^

Source: www.husqvarnacp.com.

The measurement was carried out in idle (gas trigger was not pulled), half gas (gas trigger was half full) and racing (gas trigger was fully pulled) conditions. The measurements were repeated three times in each condition with a span of 30 seconds. This procedure was done to determine the amount of sound intensity produced by the chainsaw machine and the one received by the respondents.

### Measurement of respondents’ perception toward noise

Respondents’ perception toward noise produced by the chainsaw machine was measured using a Likert scale ( Table 2). The measurement was carried out after the respondents were exposed to the sound of chainsaw in three different conditions (idle, half gas and racing). Measurement in each condition was carried out three times: without using personal protective equipment/PPE, using earmuffs (Peltor X4A, Peltor, Poland) and using earplugs (E-A-R Ultrafit 340-4002, E-A-R ^TM^, US). Respondents were then asked to describe the sound according to the Likert scale, namely. ‘very quiet’ (4.20 to 5.00), ‘not noisy’ (3.40 to 4.20), ‘quite noisy’ (2.60 to 3.40), ‘noisy’ (1.80 to 2.60), and ‘very noisy’ (1.00 to 1.80).

**Table 2.** Likert Scale of perceived noise based on weighted values.

Scale	Value interval	Noise perceived
5	4.20─5.00	Very quiet
4	3.40─4.20	No noise
3	2.60─3.40	Quite noisy
2	1.80─2.60	Noisy
1	1.00─1.80	Very noisy

### Measurement of respondents’ concentration level

The concentration levels of each respondent were measured using a questionnaire which comprised of 25 math-questions that must be filled within 10 minutes (Appendix). ^21^ The measurements were done after the respondents were exposed to the sound of chainsaw in 64 different conditions (chainsaw idle without personal protective equipment, chainsaw idle using earmuffs, chainsaw idle using earplugs, chainsaw racing without personal protective equipment, chainsaw racing using earmuffs and chainsaw racing using earplugs) for five minutes, so a total of four measurements were done and compared.

### Data analysis

Saphiro-Wilk normality test was used to ascertain the distribution of the data. Data that were not normally distributed would be analysed using non parametric test. Wilcoxon sign rank test was used to compare repeated measurements, including the sound intensity measured in respondents’ left and right ear during idle, half gas, and racing conditions, and the concentration level of each respondent without and with personal protective equipment in idle and racing conditions. The results were considered as significant if the p-value was below 0.05. All statistical analyses were performed using the Statistical Package for the Social Sciences ( SPSS, RRID:SCR_016479), version 21 (IBM ^®^ Inc., USA).

## Results and discussion

### Characteristics of the timber harvesting workers

Workers in a timber harvesting site, consisting of 20 respondents (10 chainsaw operators and 10 harvesting workers) were recruited in this study. ^22^ Harvesting workers were recruited from field foremen and heavy equipment operators working at logging site of PT. Toba Pulp Lestari Tbk, Aek Nauli Sector, and the truck driver who was in charge of transporting the harvested timber to the timber processing site. The characteristics of respondents based on age and work experience are shown in Table 3.

**Table 3.** Characteristics of respondents based on age and work experience.

No.	Characteristics of respondents	Category	*Chainsaw* operators		Harvesting workers	
			∑	%	∑	%
1	Age (years)	19–29.5	-	0	3	30
		29.5–40	5	50	4	40
		40–50.5	4	40	3	30
		50.5–61	1	10	1	10
2	Work experience (years)	≤ 3	2	20	5	50
		4–7	6	60	3	30
		8–11	1	10	1	10
		>11	1	10	1	10

Based on Table 3, majority of the saw operators were of productive age with work experience that varies from 1 year of work to the longest of 13 years of work. The respondent’s work experience expressed in years also indicates how long the workers have been exposed to noise as a consequence of their daily job up until the research began.

From interviews conducted, it was found that more than half (60%) of chainsaw operators included in this study listen to music or watch television at a high volume outside their working environment. This finding suggest that frequent exposure to noise at work may cause hearing impairment among the chainsaw operators so that they need to listen to music or television at higher volume. This fact should be a concern for company to carry out regular inspections of the operator’s hearing organs to avoid permanent hearing loss.

### Sound intensity

**Figure 1.**
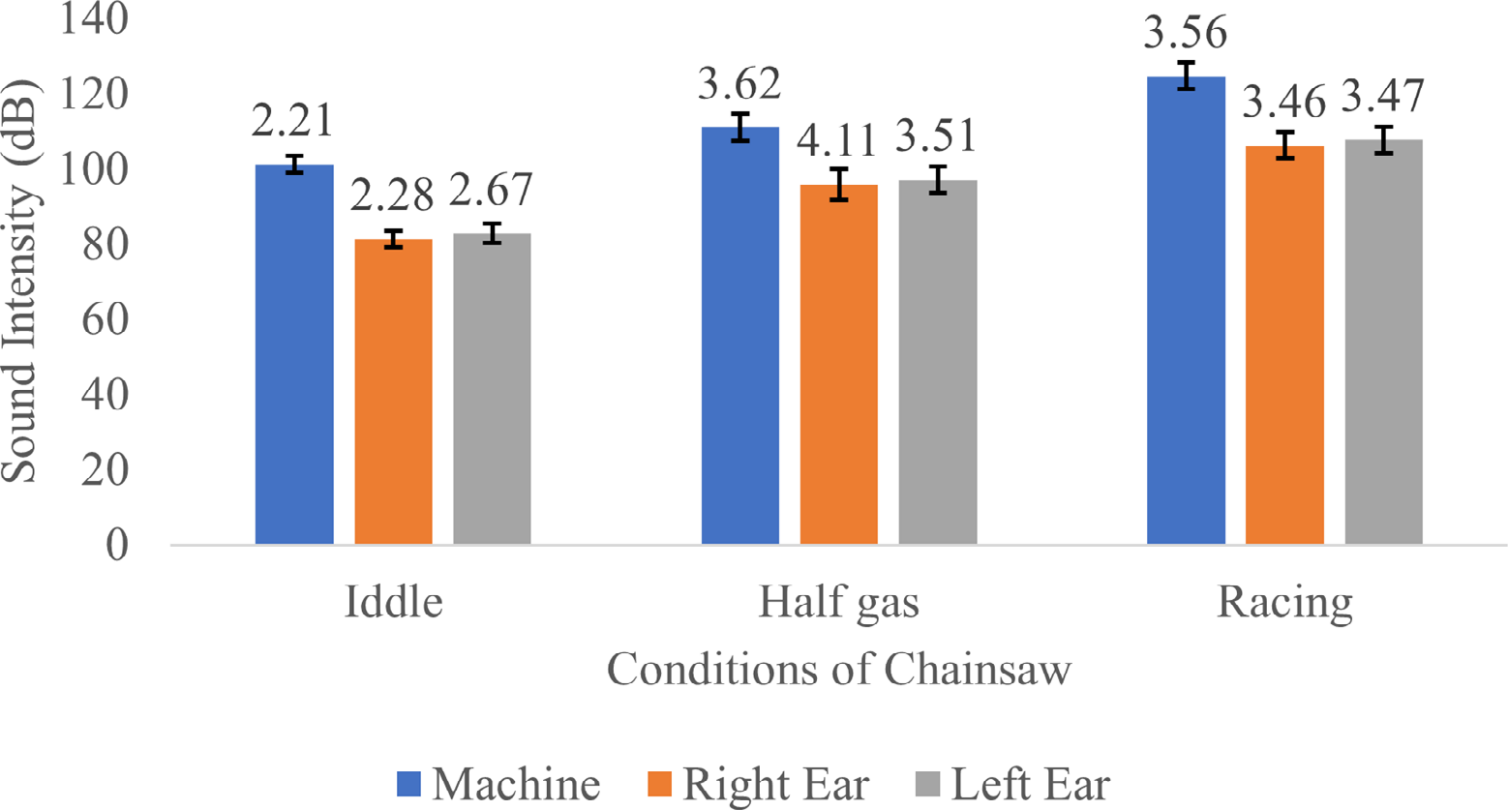
Sound intensity measured in the chainsaw and respondents’ left and right ear in idle, half gas and racing conditions. Results showed that the sound intensity received by the left ear and right ear were generally lower than those produced by the chainsaw. This is probably due to external factors including distance from the source of noise (machine) to the operator’s ear, in addition to wind and surrounding materials/environment which also reduces sound received by the ear. Meanwhile, a factor that may contributes to the difference in sound intensity received by the left ear and right ear is the hand preference of the respondent; in this study, all respondents were right-handed.

Sound intensity is one of the factors that determine the nature of a noise or the degree of hearing loss. If in a noise, the sound intensity is higher then the noise is getting louder. The sound intensities measured in the chainsaw and respondents’ left and right ear are shown in Figure 1.

In addition, the intensity of sound received by the left ear is greater than the right ear due to the distance between the sound source (chainsaw) which is closer to the left ear compared to the right ear. In this study, no detailed measurements were made of the value of the reduction in noise levels due to these external factors. This is due to the limitations of the available measuring tools.

The results of the Wilcoxson test for the noise intensity received by the respondent's right and left ears can be seen in Table 4. Based on the results of the Wilcoxon test, the intensity of noise received by the right and left ears is different in idle, half gas and racing conditions.

**Table 4.** Wilcoxon test for noise intensity received by the respondent's right and left ears.

	Wilcoxon Test p-value
Iddle on right ear and left ear	0.007
Half gas on right ear and left ear	0.011
Racing on right ear and left ear	0.028

The measurement of both sound intensity produced by the chainsaw and the intensity received by the ear at the time of timber harvesting is useful to determine how much time is allowed for working based on the ISO (International Standard Organization), OSHA (Occupational Safety and Health Association), and Indonesian standards ( Table 5).

**Table 5.** Noise threshold value standards and the allowed continuous working time.

Intensity (dB)			Working time (Hour)
ISO	OSHA	Indonesia	
85	90	85	8
-	92	87.5	6
88	95	90	4
-	97	92.5	3
91	100	95	2
94	105	100	1
97	110	105	0.5
100	115	110	0.25

Note: ISO = International Standard Organization; OSHA = Occupational Safety and Health Association.

Total effective working time of chainsaw operators at PT Toba Pulp Lestari Tbk is about 8 hours a day. From the results, when the chainsaw was turned on in idle conditions, the average sound intensity reaching the respondent’s ears was 82.11 dB ( Figure 1). Referring to Table 5, it can be concluded that in idle conditions, respondents could operate the chainsaw safely for 8 hours because the sound intensity produced by the chainsaw was still below the threshold set by the ISO (International Standard Organization), OSHA (Occupational Safety and Health Association) and Indonesian standard. Meanwhile, when the chainsaw is turned on at half gas mode, the average sound intensity received by the respondent’s ears is 96.47 dB ( Figure 1), which means that the chainsaw can be operated safely for only one hour according to ISO standards or for 4 hours and 2 hours according to OSHA and Indonesian standards, respectively. Furthermore, when the chainsaw was turned on in racing conditions, the average sound intensity received by the respondent’s ears was 106.95 dB ( Figure 1), which means that the chainsaw can be operated safely without causing any hearing impairment for 0.25 hour according to ISO standards, 1 hour according to OSHA, or 0.5 hour according to the Indonesian standard. This is in accordance with the specifications of the chainsaw machine used in the study, where the average sound produced by the machine is about 125 dB and the intensity of the sound received by the ear is about 108 dB.

During harvesting and felling activities, chainsaw operators are exposed to the noise produced by the chainsaw for approximately 4 hours every day, which means that their exposure to noise exceeds the permitted time limit set by the ISO, OSHA, and Indonesian standards.

### Respondents’ perception toward noise

Chainsaw machines cause significant noise due to the movement and friction of the components of the combustion engine which causes changes in air frequency and pressure, in addition to the movement of the chain which rotates at a high speed and rubs against the blades. Everyone’s perception of an object can be different, which may be positive or negative. The difference in perception can occur in chainsaw operators and harvesting workers to the noise they receive.

**Figure 2.**
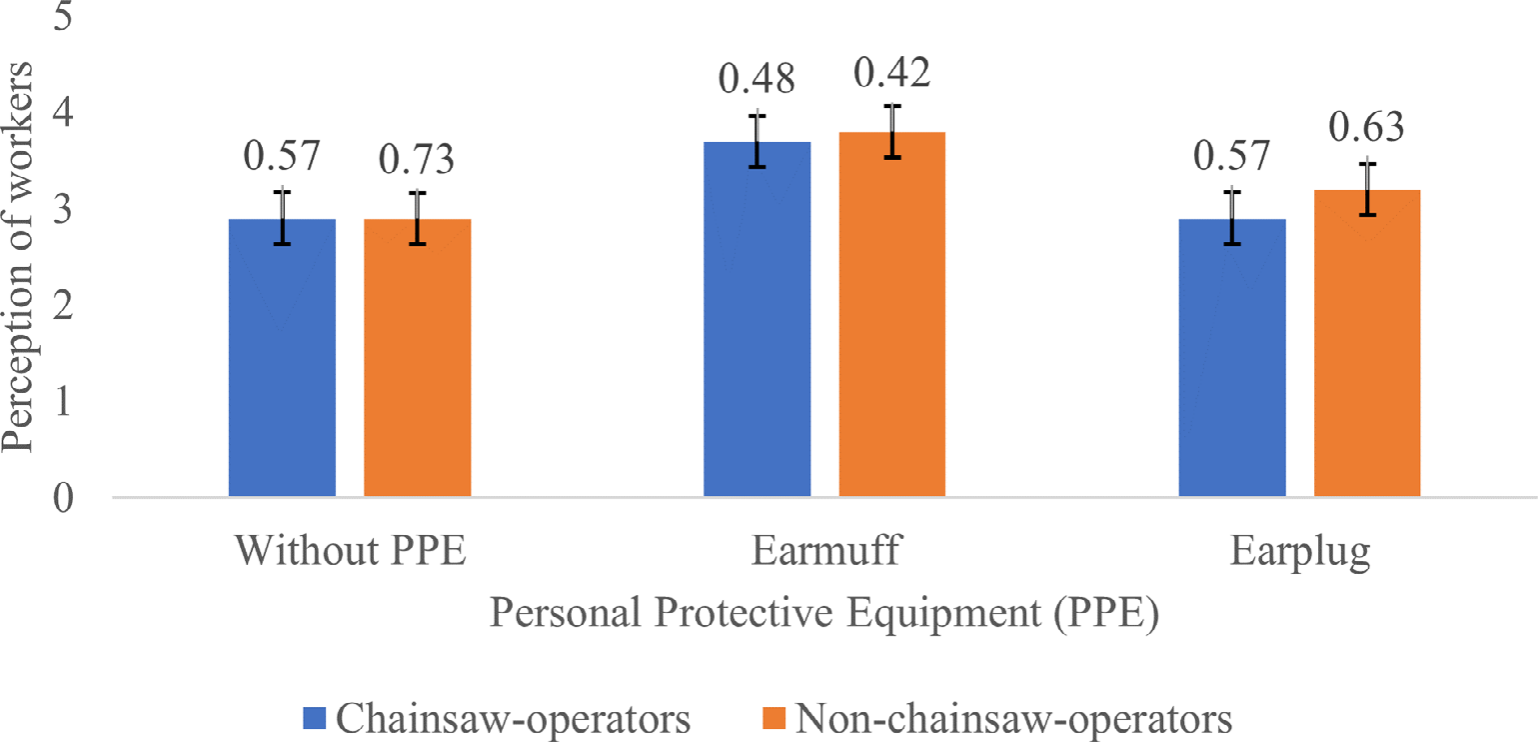
Perceptions of chainsaw operators and harvesting workers on noise produced by the chainsaw in idle condition.

**Figure 3.**
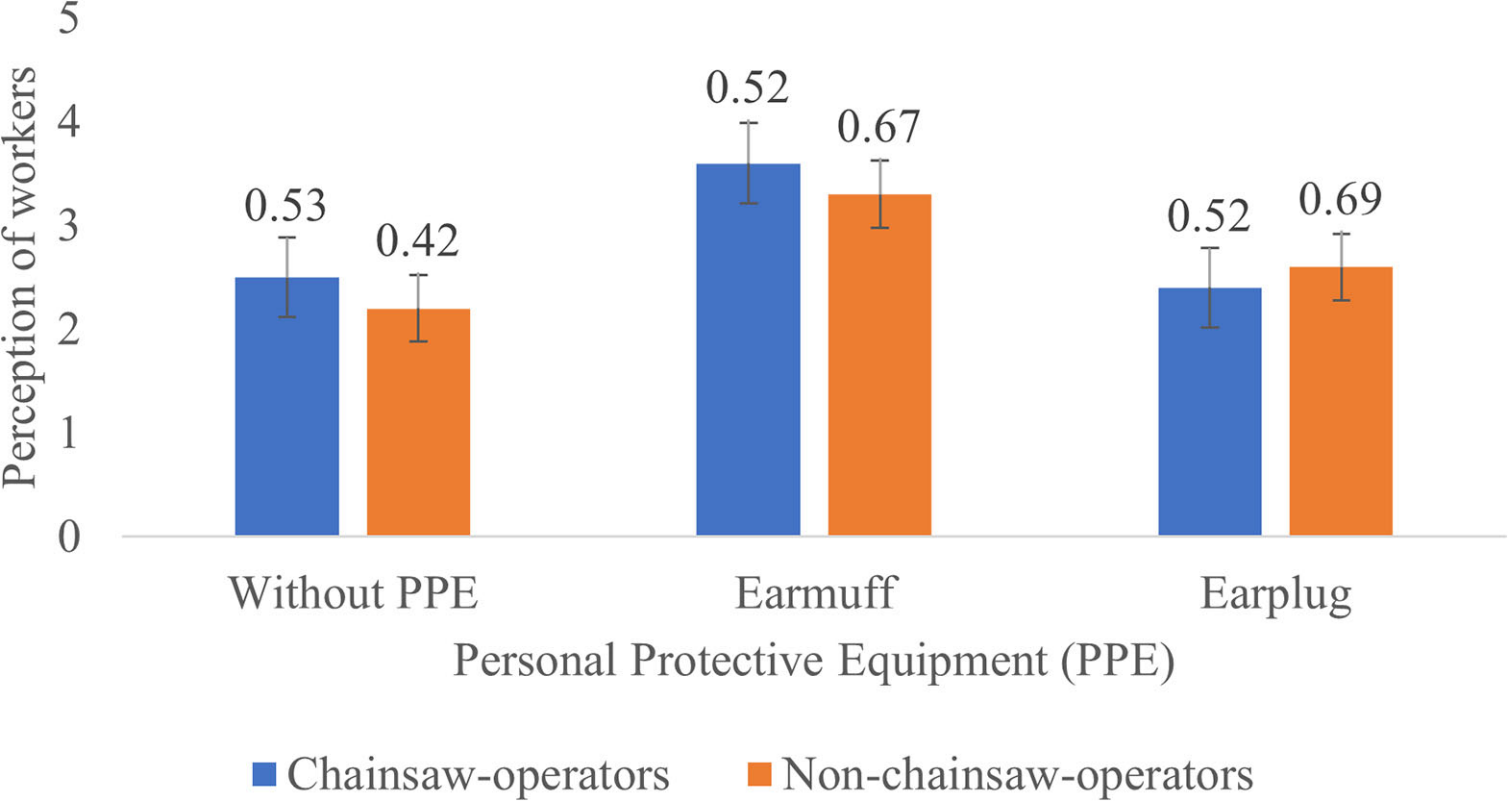
Perceptions of chainsaw operators and harvesting workers on noise produced by the chainsaw in half gas condition.

**Figure 4.**
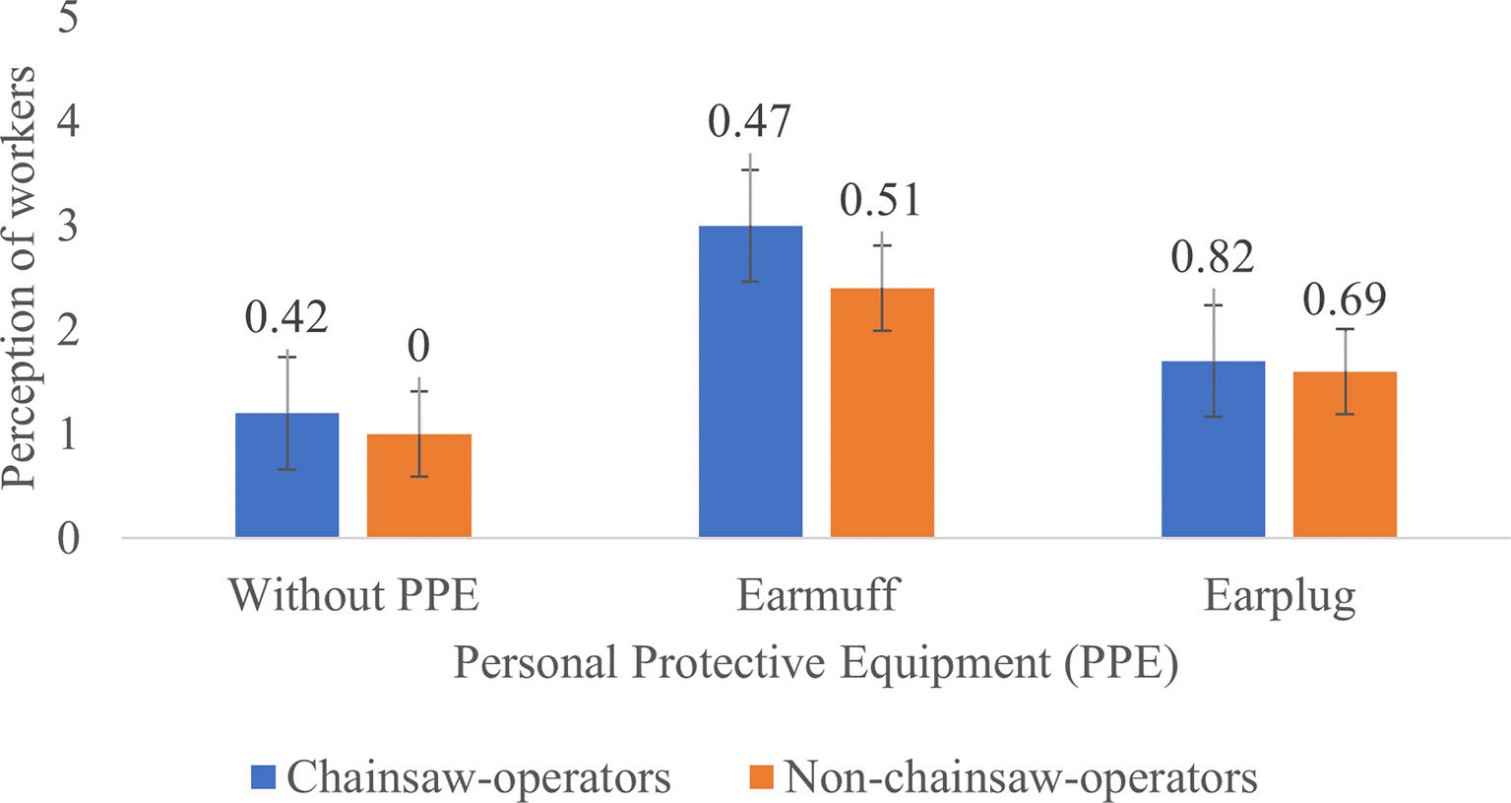
Perceptions of chainsaw operators and harvesting workers on noise produced by the chainsaw in racing condition.

Figures 2 to 4 show the different perceptions of chainsaw operators and harvesting workers on chainsaw noise without using PPE, using earmuffs and earplugs, in idle, half gas, and racing conditions, respectively. Without using PPE, both chainsaw operators and harvesting workers consider the noise produced by the chainsaw in idle mode as “quite noisy”. After using earmuffs, both chainsaw operators and harvesting workers consider the noise as “not noisy”. Both chainsaw operators and harvesting workers consider the noise as “quite noisy” after using earplugs ( Figure 2).

During half gas condition without the use of PPE, both chainsaw operator and harvesting workers consider the noise as “noisy”. After using earmuffs, the perception of the chainsaw operators was improved to “not noisy” while the perception of harvesting workers slightly improved to “quite noisy”. Then when using earplug both chainsaw operators and harvesting workers considered the noise as “noisy” ( Figure 3).

When the chainsaw was in racing condition without the use of PPE, the chainsaw operators and harvesting workers considered the noise as “very noisy”. After using the earmuffs, the perception of the chainsaw operator turned to “quite noisy” while the perception of the harvesting workers was slightly improved to “noisy” and after using the earplugs the perception of the chainsaw operators and the harvesting workers was turned to “very noisy” ( Figure 4). This result was contrary with the statement from the foreman who said that the chainsaw operators did not find the machines noisy or feel disturbed because they were already accustomed to the sound of the chainsaw.

Based on Figures 2– 4, Noise intensity (dB) tends to increase starting from the chainsaw idle, half gas and racing conditions. The higher the engine speed, the more noisy it tends to be, so that the higher the noise, the smaller the respondent's perception or the respondent feels the noise is very noisy (Likert scale in Table 2). Figures 2- 4 also show that there are differences in the perception of chainsaw operators and harvesting workers. However, both chainsaw operators and harvesting workers have the same perception trend: the higher the sound intensity, the more it considered as noisy; and the more disturbed the respondents were because the disturbance due to sounds are influenced by several factors, including loudness perception. ^23^

The difference in the perception when using earplugs and earmuffs occurs because the earmuff reduction power is stronger than the earplugs. Earmuffs can reduce noise pressure around 25-40 dB, while earplugs can reduce noise pressure around 8-30 dB. ^24^ It depends on whether or not the respondent loosens the earplugs. ^25^ Similar study results from Yovi and Suryaningsih ^21^ showed that the chainsaw operators and harvesting workers have a significantly different perception on the chainsaw sounds in each chainsaw mode (idle, half-gas, racing) and when using or not using ear protectors. This difference might rise because different respondents may react differently to the same stimulus/circumtances.

Unlike earplug which is inserted to the ear canal, earmuffs are designed to be worn over the ears. This allows the earmuffs to be worn even when there is an infection in the ear and can be provided in one size. Due to their size, earmuffs will not easily be lost, and their use can be monitored because of its visibility from a distance. ^26^ These ear protectors are usually used for protection up to 110 dB. ^24^ The disadvantage is that they can be uncomfortable for prolonged use in hot environments and interferes with the use of other protective equipment, such as goggles. ^26^ Meanwhile earplugs, being small and lightweight, tend to be more comfortable and easy to combine with other protective equipment such as hats and goggles, but because it needs to be inserted to the ear canal, it is more difficult to monitor the use of earplugs compared to earmuff and requires special fitting instructions. ^26^ This type of ear protection device is usually used for protection up to 100 dB. ^15^

It was also found that at the research site, all chainsaw operators did not use earmuffs or earplugs as personal protective equipment because it was not provided by the contractor and respondents did not know about personal protective equipment such as earmuffs or earplugs. Based on the results of interviews with all chainsaw operator respondents, in terms of the comfort of wearing earmuffs and earplugs, respondents considered earmuffs as more comfortable to use than earplugs because it offers higher noise reduction. In fact, chainsaw operators are actually willing to use PPE such as earmuffs or earplugs if it was provided by the contractor.

### Respondents’ concentration level assessment

Respondents’ concentration was assessed by ignoring pre-existing conditions that might affect the concentration levels, such as respondent’s chronotype, sleep deprivation, or any substance/drug intake (e.g caffeine). ^27^ Wilcoxon test result on the concentration level of the respondents before and after wearing PPE in idle and racing conditions are shown in Table 6.

**Table 6.** Wilcoxon test results in the concentration of wood harvesting workers.

	p-value	
	Chainsaw operators	Harvesting workers
Idle with PPE and without PPE	0.070	0.007
Racing with PPE and without PPE	0.053	0.004

Note: PPE = personal protective equipment.

Table 6 shows that based on the Wilcoxon Signed Rank Test, the concentration of chainsaw operators in idle conditions with PPEs and without PPEs and in racing conditions with PPEs and without PPEs with sig 0.070 and 0.053, respectively. This shows that the sig 0.070 and 0.053 are greater than 0.05 (5% error rate), so it can be concluded that there is no significantly difference in the concentration of chainsaw operators when idling with PPEs and without PPEs and when racing with PPEs and without PPEs. Table 6 shows that the concentration power of harvester workers in idle conditions with PPE and without PPE and racing conditions with PPEs and without PPEs with sig values of 0.007 and 0.004 respectively. This shows that the sig values of 0.007 and 0.004 are smaller than 0.05 (5% error rate), so it can be concluded that there is significantly difference in the concentration of chainsaw operators when idling with PPEs and without PPEs and when racing with PPEs and without PPEs. The use of PPE, which aimed to reduce the noise received by the respondents, did not show a significant effect on the concentration level of chainsaw-operators, both in idle and racing conditions. Wilcoxon test result shows that the concentration level of chainsaw-operators remain statistically the same before and after using PPE (p>0.05). Thus it can be concluded that the chainsaw operators were not bothered by the noise of the chainsaw or were used to the noise, and in this research, it does not give significant effects to the level of concentration of chainsaw operators in both conditions. Meanwhile Baiquni ^28^ stated that most of the operators do not feel any disturbance due to noise to themselves so that this can be used as a guide that the operator is immune to noise.

Based on the results of interviews with the cutting foreman, the majority of chainsaw operators do not feel the chainsaw is noisy when turned on. This is contrary to the results of interviews which show that chainsaw operators did actually feel the chainsaw was noisy when turned on in the racing conditions, therefore, in this study, chainsaw operators were exposed to the noise in idle and racing condition, both with PPE and without PPE. The result does not show a significant differences because although they considered the condition as noisy, chainsaw operators does not experience impaired concentration power when the chainsaw is turned on. This is contrary with the harvesting workers who cannot concentrate when noise exists (p<0.05).

Wilcoxon test results shows that noise did interfere with the concentration level of harvesting workers operators when the chainsaw was turned on in the idle and racing conditions. The concentration level of harvesting workers differ significantly before and after using PPE (p<0.05). This result indicated that the noise is disturbing to the harvesting workers, probably because they were not accustomed to the noise, and the use of PPE helped them to concentrate.

Noise, which includes sound produced by human activities, has been intensively studied for its detrimental effects on human comfort, health, and productivity. ^29^ Physical workload combined with noise intensity that exceeds 85 dB(A) for 8 hours of work could lead to fatigue symptomps such reduced concentration, physical exhaustion, dizziness and others. ^30^^,^^31^ Yovi *et al.* ^21^ stated that noisy environmental conditions can cause the operator to feel tired and lose their concentration easily. Therefore, one strategy to control noise exposure is for the company to provide replacement workers when the noise exposure has exceeds the allowed time limit and provide them with the necessary PPE.

Other strategies, ranging from engineering approaches that aim to reduce the noise by adding protective equipment to the machines, to substitution or elimination of the noise source, may be applied to control noise exposure in the industrial forest plantation. Companies may consider to substitute their machinery with ones that produce less noise. From the literature, it was found that the water-cooled engine (more cylinder four-stroke one) is less noisy than the air-cooled one-cylinder two-stroke combustion engine (low weight). According to Neri *et al*., ^32^ there are differences in noise levels between Li-ion batteries and electric chainsaws. The study showed that Li-Ion battery powered chainsaw emitted lower noise and vibrations compared to wired chainsaw; but in general, these two chainsaws were better then the endothermic chainsaw in terms of both noise and vibrations they emitted. Thefore, the use of battery-powered chainsaws may decrease the exposure to noise and onset of hand-arm vibrations when compared with the use of combustion chainsaws. ^33^

A study done by Wojtkoviak *et al*. ^34^ showed that lubricating the chainsaw’s cutting system with oil may help to reduce the noise generated by the chainsaw; even when they have similar cutting elements and used under identical condition. The noise reduction varies with different types of oil; the use of vegetable oil as lubricant resulted in the lowest noise emission. Skarzynski and Lipinski ^35^ found that a higher noise level is generated during cross-cutting with the upper side of the guide bar and that kerf height affects significantly the level of emitted noise.

Since the study was done in outdoor setting, limitations of this study mainly come from inability to control external factors, such as wind that may reduces the sound intensity received by respondents. Other than noise, there are also several factors that may affect concentration level of respondents, which were overlooked in the study. These factors include chronotype of each respondent, sleep adequacy, consumption of drugs or other substance.

## Conclusions

During harvesting and felling activities, chainsaw operators are exposed to the noise produced by the chainsaw for approximately 4 hours every day, which means that their exposure to noise exceeds the permitted time limit set by the ISO, OSHA, and Indonesian standards. The noise did not show significant effects on the power concentration of the chainsaw operators. Thus it can be concluded that the chainsaw operators do not feel disturbed by chainsaw noise or are accustomed to the noise. However, the noise produced by the chainsaw disturbs the concentration of the harvesting workers. This is thought to be due to chainsaw operators being accustomed to exposure to disturbing sounds whereas harvesting workers are not. Therefore, noise control measures are still needed to ensure the safety of sound intensity in achieving occupational safety for the workers, especially the harvesting workers.

## Data availability

### Underlying data

Zenodo: Noise Intensity and Its Impact on The Perception and Concentration Level Among Forest Harvesting Workers in Industrial Forest Plantation, North Sumatera, Indonesia. https://doi.org/10.5281/zenodo.6423524. ^22^

This project contains the following underlying data:

• Raw data of noise intensity; raw data of perception dan concentration of workers.xlsx (raw data of noise intensity and raw data of perception and concentration scores of workers)

Zenodo: Noise Intensity and Its Impact on The Perception and Concentration Level Among Forest Harvesting Workers in Industrial Forest Plantation, North Sumatera, Indonesia. https://doi.org/10.5281/zenodo.6579109. ^20^

• Blank informed consent form (English); Blank informed consent form [english].pdf
• Interview Guide; Interview Guide.pdf

Data are available under the terms of the Creative Commons Attribution 4.0 International license (CC-BY 4.0)
