# Supplementary material for: Effects of noise intensity on concentration levels of chainsaw operators and harvesting workers in industrial forest plantation, North Sumatera, Indonesia
Source: F1000Res. 2024 Jan 4;11:627. Originally published 2022 Jun 8. [Version 3] doi: 10.12688/f1000research.114592.3 (PMC10425697; doi:10.12688/f1000research.114592.3)
Supplement: Peer review history

# Reviewer report: Reviewer response for version 3

**DOI:** 10.5256/f1000research.160648.r235515

**Published:** 2024-11-21

**Authors:** Saliha Unver

**Affiliations:** 1 Karadeniz Teknik Üniversitesi, Trabzon, Turkey

The article examined the noise levels exposed to chainsaw operators and harvest workers working nearby when the chainsaw was in idle, half-throttle, and racing states. The article is interesting in that it evaluates the noise exposure of both operators and harvest workers working in wood harvesting and considers the chainsaw operation at different scales.

Is the work clearly and accurately presented and does it cite the current literature?

Yes

If applicable, is the statistical analysis and its interpretation appropriate?

Partly

Are all the source data underlying the results available to ensure full reproducibility?

Partly

Is the study design appropriate and is the work technically sound?

Partly

Are the conclusions drawn adequately supported by the results?

Partly

Are sufficient details of methods and analysis provided to allow replication by others?

Partly

Reviewer Expertise:

Forest Ergonomy, Occupational Safety and Health, Forest Harvesting

I confirm that I have read this submission and believe that I have an appropriate level of expertise to confirm that it is of an acceptable scientific standard.

# Reviewer report: Reviewer response for version 2

**DOI:** 10.5256/f1000research.151826.r191956

**Published:** 2023-08-23

**Authors:** Saliha Unver

**Affiliations:** 1 Karadeniz Teknik Üniversitesi, Trabzon, Turkey

Thank you for your article. This article is a important topic as it provides information about the occupational safety and health of harvesting workers and chainsaw operators. It provides information about chainsaw operators and forest harvesting workers' perception to the noise intensity of the chainsaw and how it affects workers' concentration. However, I have some concerns about some issues that were not taken into account in the research, the lack of adequate description of what was done and the narrative organization of the paper.

**Title**:

- It should be reorganized to reflect the main purpose of the study. Suggestion: " Effects of noise intensity on concentration levels of chainsaw operators and harvesting workers"

**Abstract**:

- Abstract section should be rewritten after the paper has been edited according to the reviewers' suggestions.
- Objective would be appropriate to specify the location of the study (in North Sumatera, Indonesia).
- The term "non-chainsaw operators" could be replaced by "harvesting workers".
- "... the concentration of workers..." in the study objective section is not an adequate description. The study determines the effects of noise on the concentration levels of both chainsaw operators and non-chainsaw operators. This should be written clearly.
- In the methods: In the first sentence mentioned as PPE use, it should be written which PPEs (earmuffs, ear plugs and without PPE).
- Results: Reliability levels determined as a result of Wilcoxon test should be stated numerically and in %.
- Conclusions: In the term "...chainsaw machine..." can be delete "machine".
- A comment could be made as to why the concentration of harvesting workers is affected by the noise of the chainsaw while the operators are not.
- The study suggested that noise control measures should be taken for harvesting workers. It would be more appropriate to suggest explicitly which measures can be taken in this study whose subject is direct noise exposure.
- Keywords: "noise level, concentration of workers, perception toward noise, chainsaw operator, forest harvesting worker" can be used.

**Introduction**

- “The permissible noise threshold" must be clearly stated in which standard it is given and how many dB it is.

**Methods**

- In the sentence mentioned as the use of PPE, it can be written which PPEs in the parentheses (Earmuffs, earplugs and without PPE).
- It would be appropriate to explain in a sentence how sound intensity is measured with a sound intensity meter. Where and how was the sound meter placed and measured?
- How many scales were there on the Likert scale (3 or 5)? Please write the meaning of the scale (1: ...; 2: ...). How many questions were used to determine perception? The questions should be written.
- What is the overall difficulty level of the 25 math questions? Could noise exposure as well as workers' level of education have an impact on the time workers spend answering the questions?
- It should be made clear the Wilcoxon sign rank test was used to determine between which the parameters relationship.

**Ethical consideration**:

- "Following the explanation about the nature of the study, written informed consent was obtained from each participant." - What is included in the written informed consent can be briefly stated.

**Population**:

- The term "Target groups" can be used instead of "Population".
- "The population in this study was all workers at PT" where the explicit spelling of PT is not given in the previous part of the text. The first time it is used, it should be spelled out and abbreviated in parentheses. In subsequent uses in the text, the direct abbreviation can be used.
- It was mentioned that it was used saturated sampling technique. All members of the population are used as samples in saturated sample technique. Were all workers at PT only 10 chainsaw operator and 10 non-chainsaw operators? It should be explained in a sentence.

**Study design and groups**:

- It is said that workers are exposed to noise by operating the chainsaw as idling, gas, racing. Is the chainsaw idle while it is being throttled or is any tree being cut down? Can the level of disturbance vary between idling and tree felling?
- It should be clearly stated whether the operators were all exposed to the sound of the same model chainsaw during the measurements and what the model of the chainsaw was.
- "According to previous study, during this range of time, healthy individual..." What is meant by healthy individual?

**Respondent Interview**

- The chainsaw model used in the study (Husqvarna 365 chainsaw) should be mentioned before Table 1. The specifications of Husqvarna 365 chainsaw should be given in Table 1.
- Reference should be added to Table 1. The new reference should also be added to the References section.
- Table 1 should be placed after the place where it is referenced in the text.

**Measurement of sound intensity**:

- The duration of the measurements is not specified. 1 day? 1 hour? During 1 tree cutting period? Workers' perception of noise depends not only on the level of noise exposure but also on the duration of noise exposure. For this reason, the duration of the measurements should be clearly stated, and the reason written down.
- "Sound intensity measurement on the chainsaw machine was done by placing the sound level meter approximately 5-10 cm from the chainsaw." It should be clearly stated where and how the sound level meter was placed it 5-10 cm away from the chainsaw.
- When determining the exposure of non-chainsaw operator to chainsaw noise, the distance of the non-chainsaw operator from the chainsaw was not taken into account. However, proximity to the noise source is a parameter that affects the noise level and perception levels to which the person will be exposed. For this reason, the distances of the non-chainsaw operators subject to the study to the chainsaw should have been specified and their relationship with perception levels should have been evaluated.
- Tables and figures should be placed immediately after the place where they are referenced in the text.
- It is not stated in the "Method" section that measurements were made on the right and left ears of the operators and workers. It should be clearly stated with which measuring instrument, when (while the chainsaw was running? After the chainsaw was switched off?) and how the measurements were made.

**Measurement of respondents' perception toward noise**

- 'very quiet' (4.20 to 5.00), 'not noisy' (3.40 to 4.20), '...”. These classes are already described in Table 2 so do not need to be written.
- Reference should be made to Table 2.
- "The measurement was carried out after the respondents were exposed to the sound of chainsaw in three different conditions (idle, half gas and racing)...Respondents were then asked to describe the sound according to the Likert scale". Did they describe each noise separately or after all the noise exposures were completed? It is not clear.
- How many questions were asked to determine perception to workers? The questions should be specified.
- “…(1.chainsaw idle without personal protective equipment, 2.chainsaw idle using earmuffs, 3.chainsaw idle using earplugs, 4.chainsaw racing without personal protective equipment, 5.chainsaw racing using earmuffs and 6.chainsaw racing using earplugs) for five minutes, so a total of **four** measurements were done and compared.” Why did not you write half gas position of chainsaw? I think that it should be nine measurements, not four. [3 PPE (without PPE, earmuffs, earplugs) x 3 chainsaw (idle, half gas, racing)] = **9**
- Were the 25 math questions administered after all the exposures were completed or while the chainsaw was operating in "idling, half throttle and racing conditions"? Were they applied separately after the exposure in each condition? If administered separately, were the same questions asked in the concentration assessments made by solving math questions? If the same questions were asked, the fact that the operator and non-operator workers had seen the questions before would not give a correct result.
- How were the math questions selected for the workers? The general difficulty level of the questions should be specified. Otherwise, the question arises that the noise exposure as well as the education level of the workers may have an effect on the time taken by the workers to answer the questions.
- The 25 math-questions can be listed in an appendix.

**Results and Discussion**

- I suggest that more references to enrich the discussion of the results can be used.
- The Discussion section should include references directly related to the results of this study. The results of the references should be compared with the results of this study. References that give general information or contain results about measurements not performed in this study should not be used in this section.

**Characteristics of the timber harvesting workers**

- Table 3 should follow the first paragraph.
- Among the demographic characteristics of the workers, their gender should be mentioned. If they are all male, it should also be indicated.
- I would suggest that after describing the Likert Scale, the corresponding ranges be added in parentheses.
- It is not clear what is meant by "...productive age...". The age range of the classification made in this way should be written in parentheses and reference should be made to where this classification is taken from.
- "The respondent's work experience expressed in years also indicates how long the workers have been exposed to noise as a consequence of their daily job up until the research began." This is not exactly the correct interpretation. Has he/she done this job continuously for the whole year of experience? The worker may have taken a break. How many days a year, how many days a month? Or how many hours a day the operator cuts trees. For harvesting workers, it also depends on how much of the day they are working in an environment with chainsaws and their proximity to the sound source.
- No vibration measurements were carried out within the scope of the study. Therefore, it is not necessary to include the results of Morata *et al.* and Pyykkö *et al.* on the effects of vibration in the discussion section.
- Figure 1 should be placed after the citation.
- In the paragraph after Figure 1 it is stated "Results showed that the sound intensity received by the left ear and right ear were generally lower than those produced by the chainsaw." It should be mentioned in the method section that the sound produced by the chainsaw was measured. The results on this subject should be given in the Results section.

**Sound intensity**

- "During harvesting and felling activities, chainsaw operators are exposed to the noise produced by the chainsaw for approximately 4 hours every day," if identified in this study, how it was identified should be mentioned in the Method section. If it is not the result of this study, it should be cited if quoted. Does the whole job take 4 hours? How many hours of this time is the saw idling and how many hours is the saw racing? Accordingly, how many dB of noise is the worker exposed to on average during 4 hours of work? These should be stated and interpreted.
- Rukat *et al *compared the noise and vibration levels of 2 different saws. Since this study did not have findings on different types of chainsaws or vibration measurements, it is not appropriate to use it in the discussion section.
- Indrayani *et al.* provides general information and does not discuss the results of this study. It is better used in the Introduction section.

**Respondents' concentration level assessment**

- Last sentence of this section: "These factors include chronotype of each respondent, sleep adequacy, consumption of drugs or other substance" repeated in the first sentence of the same section.

**Conclusion**

- In this study, the safety levels of sound intensity to which workers are exposed according to various standards (ISO, OSHA, Indonesian standard) are also compared in Result and Discussion section. The results of these comparisons should also be mentioned in the Conclusion section.

Is the work clearly and accurately presented and does it cite the current literature?

Yes

If applicable, is the statistical analysis and its interpretation appropriate?

Partly

Are all the source data underlying the results available to ensure full reproducibility?

Partly

Is the study design appropriate and is the work technically sound?

Partly

Are the conclusions drawn adequately supported by the results?

Partly

Are sufficient details of methods and analysis provided to allow replication by others?

Partly

Reviewer Expertise:

Forest Ergonomy, Occupational Safety and Health, Forest Harvesting

I confirm that I have read this submission and believe that I have an appropriate level of expertise to confirm that it is of an acceptable scientific standard, however I have significant reservations, as outlined above.

# Reviewer report: Reviewer response for version 2

**DOI:** 10.5256/f1000research.151826.r187528

**Published:** 2023-08-14

**Authors:** Mohamad Siarudin

**Affiliations:** 1 Research Center for Ecology and Ethnobiology, National Research and Innovation Agency (BRIN), Bogor, Jawa Barat, Indonesia

Thank you very much for your revised version of the manuscript. In general, I found this manuscript has been significantly adjusted incorporating Reviewers' comments.

 Congratulations.

Is the work clearly and accurately presented and does it cite the current literature?

Yes

If applicable, is the statistical analysis and its interpretation appropriate?

Partly

Are all the source data underlying the results available to ensure full reproducibility?

Partly

Is the study design appropriate and is the work technically sound?

Partly

Are the conclusions drawn adequately supported by the results?

Partly

Are sufficient details of methods and analysis provided to allow replication by others?

Yes

Reviewer Expertise:

Forest management; forest product technology; agroforestry; carbon accounting

I confirm that I have read this submission and believe that I have an appropriate level of expertise to confirm that it is of an acceptable scientific standard.

# Reviewer report: Reviewer response for version 2

**DOI:** 10.5256/f1000research.151826.r187527

**Published:** 2023-07-24

**Authors:** Vasiliki Dimou

**Affiliations:** 1 Democritus University of Thrace, Komotini, Greece

The revisions are appropriate and I do not have any new comments.

Is the work clearly and accurately presented and does it cite the current literature?

Yes

If applicable, is the statistical analysis and its interpretation appropriate?

Partly

Are all the source data underlying the results available to ensure full reproducibility?

Partly

Is the study design appropriate and is the work technically sound?

Yes

Are the conclusions drawn adequately supported by the results?

Partly

Are sufficient details of methods and analysis provided to allow replication by others?

Partly

Reviewer Expertise:

Forest Products Harvesting and Forest Ergonomics

I confirm that I have read this submission and believe that I have an appropriate level of expertise to confirm that it is of an acceptable scientific standard.

# Reviewer report: Reviewer response for version 1

**DOI:** 10.5256/f1000research.126338.r170754

**Published:** 2023-06-05

**Authors:** Mohamad Siarudin

**Affiliations:** 1 Research Center for Ecology and Ethnobiology, National Research and Innovation Agency (BRIN), Bogor, Jawa Barat, Indonesia

Thank you for your interesting article. This article seems good in originality and clear in the problem addressed. It provides information on how the noise intensity affects the perception and concentration of the forest harvesting workers. However, I have some concerns on different sections of the manuscript.

**Introduction**

- Well organized. Clear research objective, however, I would suggest including the safety aspect of sound intensity in the objective and conclusion.

**Method**

- You mention that the sampling method was a saturated sampling technique. Does it mean that all of the population was included in the survey? How many populations? Please mention it.
- Please add a brief reason for the “right-handed” respondent criteria.
- I found redundancies in describing idle, gas, and racing.

**Result**

- Please avoid using a single sentence in one paragraph (please also recheck throughout the paper).
- Figure 1: It would be nice if you can show the deviation standard (among 3 replications) in the figure.
- Please check your referring (Table 1? Should be Table 2?) in the Sound intensity subsection, paragraph 4 ^th^.
- I would suggest more references to enrich the discussion of the findings in the sound intensity subsection.
- Figure 2-4: the use of scale in the Y-axis is not clear and is not described in the method. In the description, you use categorical data: very noisy, noisy, quite noisy. Please make it clear and consistent in the methods, figure, and discussion.
- Why does the perception between Figure 2 (idle), 3 (half-gas), and 4 (racing) tend to decrease in scale? In my understanding, the sound should tend to increase.

**Conclusion**

- The conclusion seems to answer the objective of the study, which focuses on the concentration of the workers. However, this study also covers the safety aspect of sound intensity by comparing it to several standards, as well as the perception of the workers (that appears in the title). In my opinion, this safety aspect is interesting and could be included in the study objective and conclusion.

Is the work clearly and accurately presented and does it cite the current literature?

Yes

If applicable, is the statistical analysis and its interpretation appropriate?

Partly

Are all the source data underlying the results available to ensure full reproducibility?

Partly

Is the study design appropriate and is the work technically sound?

Partly

Are the conclusions drawn adequately supported by the results?

Partly

Are sufficient details of methods and analysis provided to allow replication by others?

Yes

Reviewer Expertise:

Forest management; forest product technology; agroforestry; carbon accounting

I confirm that I have read this submission and believe that I have an appropriate level of expertise to confirm that it is of an acceptable scientific standard, however I have significant reservations, as outlined above.

# Response

**Published:** 2023-06-19

**Authors:** Muhdi Muhdi

**Affiliations:** Universitas Sumatera Utara, Indonesia

Thank you very much for reviewing our manuscript. We have revised the manuscript according to your comments and suggestions. Thank you for kindly.

# Reviewer report: Reviewer response for version 1

**DOI:** 10.5256/f1000research.126338.r174078

**Published:** 2023-06-01

**Authors:** Vasiliki Dimou

**Affiliations:** 1 Democritus University of Thrace, Komotini, Greece

Thank you very much for giving me the opportunity to evaluate this article. The purpose of the article is interesting and very practical. The authors describe the exposure of the forest harvesting workers in an industrial forest plantation to the noise in three different conditions (idle, half gas and racing conditions).

- The work is clearly and accurately presented and it cites the current literature but be careful in p. 4: *"The interview guide was developed by the authors to include all necessary data and no prior testing was done* ^***39***^"- Maybe the number of the bibliographic reference is 20 and not 39?
- The study design is appropriate and the work is technically sound, but be careful in p. 5 "... *chainsaw in 4 different conditions (chainsaw idle without personal protective equipment, chainsaw idle with personal protective equipment, chainsaw racing without personal protective equipment and chainsaw racing with personal protective equipment) for five minutes, so a total of **four measurements** were done and compared…" - *I wonder if the correct one is six measurements instead of four measurements, given that 'personal equipment' includes 2 conditions 'earmuffs and earplugs'?
- In the context of replication and comparison it would be good to show the main technical characteristics of the Husqarna 365 chainsaw in a table (p.4) (Engine Size/Power Hp/ Max rpm/Capacity/Weight empty.
- In addition I would suggest that the questionnaire (25 math-questions) be listed in an appendix.
- The conclusions are drawn adequately supported by the results. But be careful in p.7: "... *while the non-chainsaw operators consider it as “ **very noisy**”..." -* I wonder if the correct one is ‘quite noisy’?
- *After using earmuffs, both chainsaw operators and non-chainsaw operators consider* *the noise as “ **noisy**”..."*  - I wonder if the correct one is ‘’not noisy’’?
- I would suggest that after a description of the Likert scale, the corresponding limits should be included in parentheses e.g. ‘not noisy’ (3.40-4.20).
- In fig. 1 the unit of measurement is missing, I assume it is dB?
- In fig. 2-4, for better comparison, the axes ‘Perception of workers’ should contain the entire Likert scale, i.e. 0-5.
- p.4: "... *The structured-interview was done by **AAT **at the logging site..."* - It must be mentioned, at least for the first time, written in full in parentheses (Associate of Accounting Technicians).
- p.5-6: "... *In a previous study...Altogether, these findings emphasized the importance of using personal protective equipment to reduce the noise." - * These references are better suited to the introduction than here. At this point literature references on noise would be interesting.
- p.6: "... *Based on the Wilcoxon test result, it can be concluded that there **was no significant difference** in the sound intensity received by respondents’ left and right ear during idle, half gas, and racing condition (p<0.05)." - *Shouldn’t the right and left ear noise difference be treated numerically, because sound level is a logarithmic quantity? For example, it is stated that for sound levels that differ by 3 dB, the stronger one has twice the intensity of the weaker one, that is, the level of 85 dB is twice as loud as that of 82 dB, while two 85 dB sounds added have a level of 88 dB and not 170 dB.
- Logarithmic measurements of sound intensity must be converted into numerical measurements of sound intensity using: e ^x^ =y, where x the results in logarithmic measurements of sound intensity. So with the new numerical measurements of sound intensity ‘y’, the Wilcoxon test must be done again.
- p.6: "... *Referring to **Table 1**, it can be concluded that in idle conditions,..." - *Is Table 2 likely to be the correct one?

Is the work clearly and accurately presented and does it cite the current literature?

Yes

If applicable, is the statistical analysis and its interpretation appropriate?

Partly

Are all the source data underlying the results available to ensure full reproducibility?

Partly

Is the study design appropriate and is the work technically sound?

Yes

Are the conclusions drawn adequately supported by the results?

Partly

Are sufficient details of methods and analysis provided to allow replication by others?

Partly

Reviewer Expertise:

Forest Products Harvesting and Forest Ergonomics

I confirm that I have read this submission and believe that I have an appropriate level of expertise to confirm that it is of an acceptable scientific standard, however I have significant reservations, as outlined above.
